# Supplementary figures and images for: Budbreak patterns and phytohormone dynamics reveal different modes of action between hydrogen cyanamide- and defoliant-induced flower budbreak in blueberry under inadequate chilling conditions
Source: PLoS One. 2021 Aug 31;16(8):e0256942. doi: 10.1371/journal.pone.0256942 (PMC8407589; doi:10.1371/journal.pone.0256942)

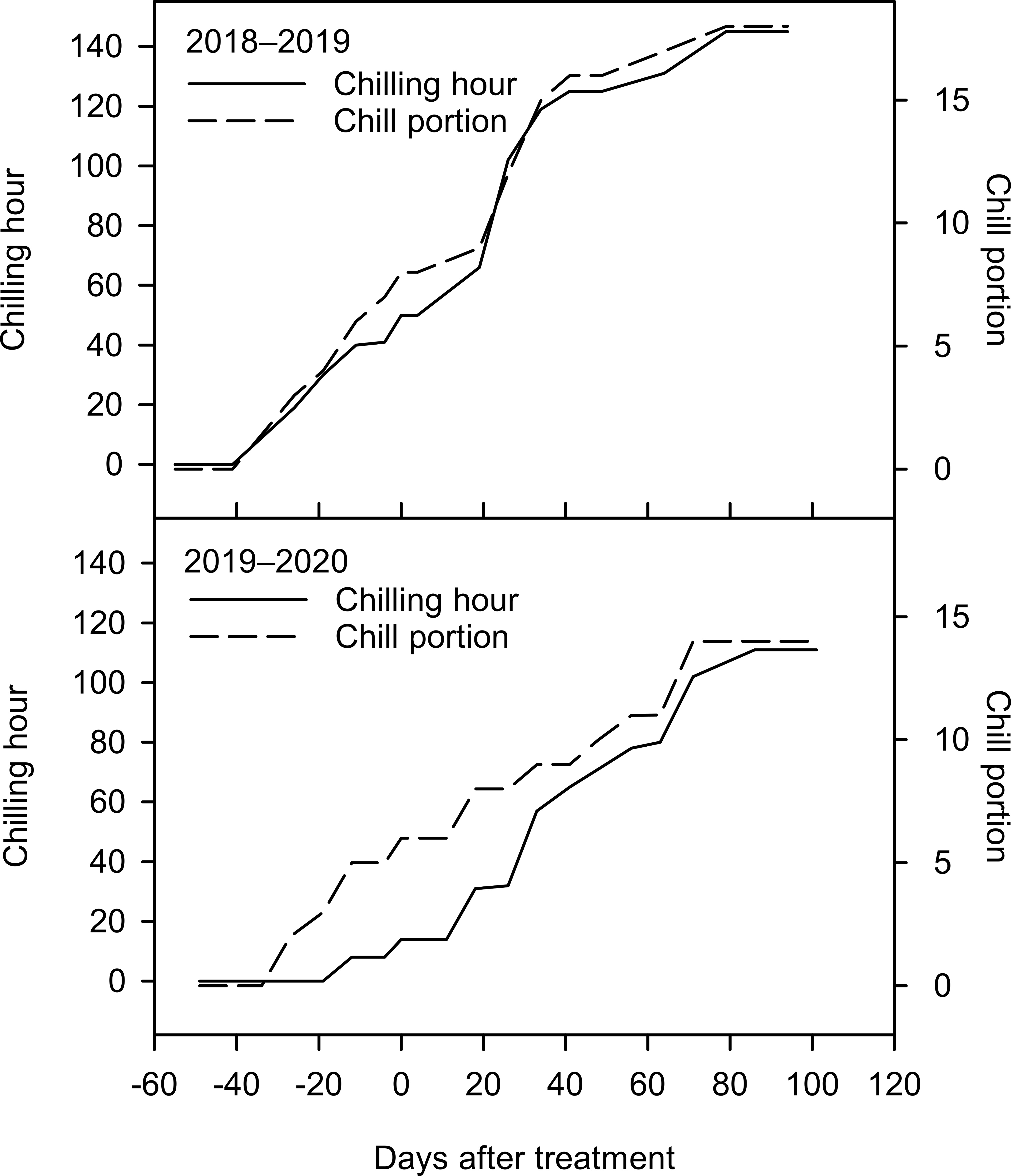

Supplement: S1 Fig — The number of accumulated chilling hours below 7.2°C recorded at the experiment site was obtained from the Florida Automated Weather Network (http://agroclimate.org/tools/chill-hours-calculator/). (TIF) [file pone.0256942.s001.TIF]

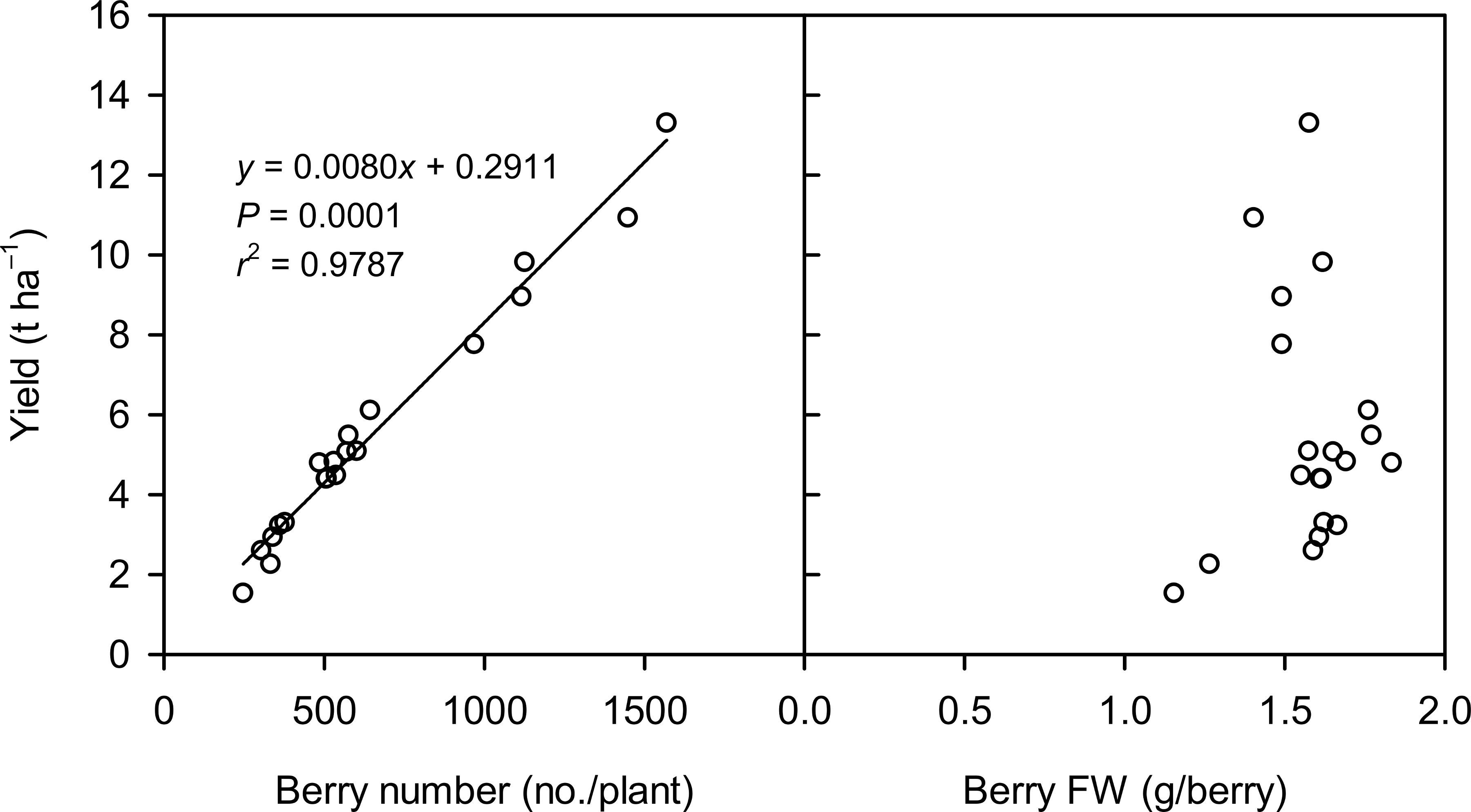

Supplement: S2 Fig — Treatments are as described in Fig 1. No line was included for the correlation between berry FW and yield because of non-significant correlation. (TIF) [file pone.0256942.s002.TIF]
