# Supplementary material for: Budbreak patterns and phytohormone dynamics reveal different modes of action between hydrogen cyanamide- and defoliant-induced flower budbreak in blueberry under inadequate chilling conditions
Source: PLoS One. 2021 Aug 31;16(8):e0256942. doi: 10.1371/journal.pone.0256942 (PMC8407589; doi:10.1371/journal.pone.0256942)
Supplement: Attachment — Submitted filename: Response to Reviewers.pdf [file pone.0256942.s003.pdf]

UF|IFAS
UNIVERSITY of FLORIDA

14625 CR 672
Wimauma, FL 33598
Tel: (813) 419-6670
http://gcrec.ifas.ufl.edu/

Gulf Coast Research and Education Center

Shinsuke Agehara
UF/IFAS Gulf Coast Research and Education Center
Wimauma, FL 33598
813-419-6583, sagehara@ufl.edu

August 17, 2021

Dear Dr. Chunxian Chen :

Thank you so much for your feedback and the opportunity to revise and resubmit our manuscript "Budbreak patterns and phytohormone dynamics reveal different modes of action between hydrogen cyanamide- and defoliant-induced flower budbreak in blueberry under inadequate chilling conditions" (PONE-D-21-21254). The suggestions and insights provided by you and the reviewers have been an enormously helpful source of perspective throughout the revision process.

Immediately following this letter, I have listed the reviewer comments and my individual responses to them, which describe how each reviewer's concern and comments were addressed in the revision. Line numbers are provided when suitable so that reviewers can immediately locate changes. We have made major revisions in the manuscript to address all the suggestions and comments by reviewers.

Thank you once again for reconsidering our manuscript for publication. We look forward to your response.

Sincerely,

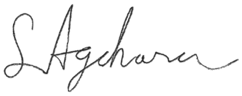

Shinsuke Agehara
Assistant Professor, Plant Physiology

14625 CR 672
Wimauma, FL 33598
Tel: (813) 419-6670
http://gcrec.ifas.ufl.edu/

Gulf Coast Research and Education Center

Additional Editor Comments:

1. The paper provided useful results and understanding for use of HC and other defoliation agents on blueberry production with insufficient chill. Experiments were well designed and data well analyzed. However, Fig. 7 appeared to be overstretched and weak. If ZS also suggestively induces the ABA-JA rapid process as HC, it would difficult to explain how the effects are different between them. It would be more convincing and reasonable the process and responses to the two agents are rather very different from each other.

ANS: Thank you for your suggestions! We think the modes of action of HC- and ZS-induced budbreak share some differences and similarities. We found that both HC and ZS increase JA production, but ZS induced JA accumulation more rapidly than HC. Therefore, we propose that ZS does not stimulate the antagonism between JA and ABA as effectively as HC (L488-490). To avoid presenting overstretched conclusion in Fig. 7, we used solid lines to demonstrate highly likely relations and dashed lines to indicate hypothetical interactions obtained from literature.

2. In addition, there are reports on substantial phytotoxicity of HC to buds and new growth in other temperate crops, which might be a lead factor of yield loss. Were there data on phytotoxicity to blueberry from those experiments? If so, it should be included in the paper.

ANS: We did these experiments in a commercial orchard and all HC treatments were performed following the instruction on Dormex's label. Because we and growers did not observe any phytotoxicity during the experimental period, we think the lead factor of yield loss was due to the difference in chilling hours between the two seasons.

Reviewers' comments:

Reviewer's Responses to Questions

Reviewer #1: This manuscript brings forward valuable information for researchers and producers of temperate fruit crops such as blueberry. The information could have been presented in a more organized manner with greater clarity however. The description of the treatments was not very clear, which made it difficult to follow the results and discussion. It was not until I got to the conclusions that I was more certain of my understanding of the manuscript findings.

1. This study is particularly important to the fruit industry. Please make every effort to make the treatment descriptions and the findings more clear.

ANS: Thank you for your comments! We have made revisions in the manuscript to address all your suggestions.

2. Line 44: Delete 'adaptation'.
ANS: Revised as suggested (L44).

14625 CR 672
Wimauma, FL 33598
Tel: (813) 419-6670
http://gcrec.ifas.ufl.edu/

Gulf Coast Research and Education Center

3. Line 66-67: Is the budbreak mentioned in these citations a result in defoliation?
ANS: In the almond study (Hegazi, 2012), defoliation data was not presented. Therefore, we decide to delete this citation. In the blackberry study (Lin and Agehara, 2021), urea, zinc sulfate and potassium thiosulfate (KTS) were sprayed as defoliants and defoliation percentage was recorded. The authors concluded that defoliation is one of many factors involving in budbreak induction (L65-67).

4. Line 103-104: Is this 1% of product or ai?
ANS: It is 1% of ai (L101, L108).

5. L113-118: Description of the experimental treatments is unclear to say the least. What are the treatments - spray volume? concentration? rates?
ANS: We've revised the paragraph to clarify the treatments in Expt. 3 (L112-115).

6. Line 137: stored at -80 C for how long?
ANS: For about six months. We've added this information (L136).

7. Line 204-208: This was previously stated in the m and m where it belongs.
ANS: Deleted as suggested (L203).

8. Line 230: Treatment produced ripened berries.
ANS: Revised as suggested (L226).

9. Line 290: Treatment may be depicted by the wrong color in the graph. Please check.
ANS: We've checked it. Can you point out which colors are wrong please? (Fig. 4C)

10. Line 357: "...chill hour increased only by 12 hours." Not sure how this was determined.
ANS: The number of accumulated chilling hours below 7.2°C recorded at the experiment site was obtained from the Florida Automated Weather Network (http://agroclimate.org/tools/chill-hours-calculator/) (Supplementary Figure 1). The difference of chill hours between 22 and 0 DAT is determined by subtracting chill accumulation at 0 DAT from chill accumulation at 22 DAT (L352-353).

11. Line 398-399: This is worded as though Florida is not a part of the United States. Please reword this.
ANS: Revised as suggested (L394-396).

12. Line 504: Please place comma after 'conditions'.
ANS: Revised as suggested (L502).
